# Risk of breast cancer and family history of other cancers in first-degree relatives in Chinese women: a case control study

**DOI:** 10.1186/1471-2407-14-662

**Published:** 2014-09-11

**Authors:** Wenbin Zhou, Qiang Ding, Hong Pan, Naping Wu, Mengdi Liang, Yaoyu Huang, Lin Chen, Xiaoming Zha, Xiaoan Liu, Shui Wang

**Affiliations:** Department of Breast Surgery, The First Affiliated Hospital with Nanjing Medical University, 300 Guangzhou Road, 210029 Nanjing, China

**Keywords:** Breast cancer, Family history, Risk factor, First-relative, Case control

## Abstract

**Background:**

Few studies have systematically reported the relationship between the risk of breast cancer and family history of other cancers. This study was designed to systematically determine the relationship between breast cancer risk and family history of other cancers in first-degree relatives.

**Methods:**

Between January 2006 and June 2011, 823 women diagnosed with breast cancer were included, and age-matched women diagnosed with benign breast disease were selected as controls. Family history of other cancers in first-degree relatives was recorded by trained reviewers. Multivariate logistic regression was applied to analyze the relationships.

**Results:**

A family history of esophagus cancer (OR: 2.70, 95% CI: 1.11 – 6.57), lung cancer (OR: 2.49 95% CI: 1.10 – 5.65), digestive system cancer (OR: 1.79, 95% CI: 1.14 – 2.79) and any cancer (OR: 2.13, 95% CI: 1.49 – 3.04) in first-degree relatives was directly associated with increased breast cancer risk. In subgroup analysis, the risk of hormone receptor positive breast cancer was increased in subjects with a family history of lung cancer (OR: 3.37, 95% CI: 1.45 – 7.82), while the risk of hormone receptor negative breast cancer was increased in subjects with a family history of esophagus cancer (OR: 6.19, 95% CI: 2.30 – 16.71), uterus cancer (OR: 6.92, 95% CI: 1.12 – 42.89), digestive tract cancer (OR: 2.05, 95% CI: 1.03 – 4.10) and gynecology cancer (OR: 6.79, 95% CI: 1.46 – 31.65). Additionally, a significant increase in breast cancer was observed with a family history of digestive system cancer for subjects 50 y and younger (OR: 1.88, 95% CI: 1.03 – 3.43), not for subjects 50 y older (OR: 1.67, 95% CI: 0.86 – 3.25).

**Conclusions:**

Breast cancer aggregates in families with several types of cancer especially for digestive system cancer. The influence of a family history of other cancers seems more likely to be limited to hormone receptor negative breast cancer.

## Background

Breast cancer is a prevalent malignant disease in the world and the leading cause of death in women [1, 2]. Plentiful risk factors have been identified, including anthropometric factors, reproductive factors, home environment, and genetic factors [3–5]. Family history of breast cancer is a key breast cancer risk factor. It provides clues as to the likelihood of a hereditary breast cancer syndrome and the need for cancer genetics, and can be used to estimate a woman’s risk in the setting of a breast cancer risk assessment model [6, 7].

Importantly, several studies have suggested that an increased breast cancer risk is observed in subjects with a family history of other cancers, including colon cancer, prostate cancer, ovarian cancer and some other cancers [8–11]. To our knowledge, few studies have systematically reported the relationship between the risk of breast cancer and family history of other cancers. Negri E and colleagues have found there was no material association between family history of cancer (breast cancer excluding) and breast cancer risk in Italy [8]. The magnitude of the association with family history varies between studies, cancer sites, countries, and state of sex and age, being generally stronger for younger probands [3, 12–15]. Thus, it is necessary and important to determine the relationship between family history of all cancers and the risk of breast cancer in different populations.

According to estrogen receptor (ER) and progesterone receptor (PR) status, breast cancer can be divided into two subgroups: hormone receptor positive and negative breast cancer. Hormone receptor positive and negative breast cancer may share disparate genetic risk factor profiles [16–19]. To our knowledge, the relationship between having a family history of other cancers and risk of subtype of breast cancer has not been reported. Additionally, previous studies have reported that patients (colorectal and lung cancer) with a family history of cancer were younger than those without a family history [20, 21]. In our country, about 40% of breast cancer patients are younger than 50 years old [22–24]. Therefore, it is important to determine the relationship between family history of other cancers and breast cancer risk among women younger than 50 years old.

Given poor reliability of the recall of cancer in second-degree relatives [8], only first-degree relatives were used in our analysis to reduce recall bias. This hospital-based case-control study was designed to systematically determine the relationship between breast cancer risk and family history of other cancers in first-degree relatives. In addition, the relationship between family history of other cancers and breast cancer risk among women younger and older than 50 years old was determined. Also, the relationship between family history of other cancers and hormone receptor positive or negative breast cancer risk was investigated separately.

## Methods

### Study population

As previously described [25], eligible women were aged 23 – 83 years, with histological confirmed breast cancer between January 2006 and June 2011 in our hospital, and 1:1 age-matched (±5 years) controls were selected in the same period, with histological confirmed benign breast disease, including fibroadenoma (316), mastopathy (97), intraductal papilloma (311), and others (99). Of these 823 controls, 311 (intraductal papilloma) were classified as benign breast disease with a low risk of developing breast cancer, and only 13 (atypical hyperplasia) were classified as benign breast disease with a high risk of developing breast cancer [26].

The present case-control study was performed with the approval of the ethics committee of The First Affiliated Hospital with Nanjing Medical University, and this study was also in compliance with the Helsinki Declaration of the World Medical Association. All patients included in this study provided written informed consent for their clinical data to be reviewed by us.

### Data extraction

The interviews of family history for all cases and controls were performed before diagnose. The clinical data were reviewed by trained interviewers. The family history of other cancers and potential risk factors were extracted from medical record review. As described previously [25], the following potential risk factors were collected: age, age at menarche, previous childbearing, menopausal status. Family history of cancers was not complete in 60 cases and 9 controls. Otherwise, age at menarche was not available in 219 cases and 154 controls; previous childbearing was not available in 144 cases and 77 controls; menopausal status was not available in 105 cases and 41 controls.

Besides, ER and PR status were reviewed and recorded. Immunohistochemical analyses on paraffin-embedded material were used to determine the ER and PR status. The cut-off positivity was 10% tumor cells for the evaluation of ER and PR. Hormone receptor positive was defined as ER positive and/or PR positive, and hormone receptor negative was defined as both ER and PR were negative. Of 823 cases, 514 were identified as hormone receptor positive, 214 were identified as hormone receptor negative, and hormone receptor status could not be identified in 95 cases.

### Statistical analysis

Median, percentiles, range, and standard deviation (SD) were analyzed for each continuous variable. Univariate and multivariate analyses were applied to estimate odds ratio (OR) and 95% confidence interval (CI) for the association between family history of other cancers and breast cancer risk. In addition, multivariate logistic regression was also applied to estimate OR and 95%CI for the association between family history of other cancers and risk of subtype-specific breast cancer, including hormone receptor positive breast cancer, hormone receptor negative breast cancer, patients diagnosed with breast cancer **≤** 50 years, and patients diagnosed with breast cancer **>** 50 years. The candidate explanatory variables in the multivariate logistic regression analysis were: age (≤30, 31-40, 41-50, 51-60, 61-70, 71-80, and ≥ 81 years), age at menarche (≤13, 14-16, and ≥17 years), previous childbearing (0, 1, 2, and ≥3) and menopausal status (premenopause, and postmenopause). The categories of age, age at menarche, and previous childbearing were described previously [25]. All variables in the multivariate logistic regression analyses were defined as grouping variables, not continuous variables. All statistical analyses were performed by using Stata version 11.0 (StataCorp, College Station, Tex). A two-sided level of significance of 0.05 was applied in all tests.

## Results

A total of 823 women with breast cancer and 823 age-matched controls were studied. Age distribution of all participants was shown in Table 1. The proportions of subjects in each age group were almost the same (*P* > 0.05). Cases and controls in this study were very well matched on age. The other basic characteristics of all included subjects were described previously [25].

**Table 1:** **Age distribution of participants in the case-control study**

Age groups	Cases, n (%)	Controls, n (%)
≤ 30 y	17 (2.1%)	17 (2.1%)
31 – 40 y	140 (17.0%)	140 (17.0%)
41 – 50 y	296 (36.0%)	298 (36.2%)
51 – 60 y	235 (28.6%)	233 (28.3%)
61 – 70 y	96 (11.7%)	93 (11.3%)
71 – 80 y	36 (4.4%)	39 (4.7%)
≥ 81 y	3 (0.4%)	3 (0.4%)
Total	823 (100%)	823 (100%)
Median	49 y	49 y
SD	10.82 y	10.82 y

The number of cases and controls with a history of selected cancers was shown in Table 2. 106 subjects were reported with a family history of selected cancers in case group and 70 subjects in control group. Of these subjects, more than 50% were reported with a family history of digestive system cancer in both groups.

**Table 2:** **Odds ratio of breast cancer according to family history of selected cancers**

Site	Cases/control (n/n)	Unadjusted OR (95% CI)	OR (95% CI) ^*^
Breast	26/16	1.76 (0.94 – 3.31))	**2.32 (1.07 – 5.03)**
Lung	21/13	1.74 (0.87 – 3.51)	**2.49 (1.10 – 5.65)**
Esophagus	18/7	**2.79 (1.16 – 6.71)**	**2.70 (1.11 – 6.57)**
Stomach	19/17	1.20 (0.62 – 2.32)	1.27 (0.61 – 2.61)
Intestine	7/8	0.93 (0.34 – 2.59)	0.91 (0.29 – 2.80)
Liver	10/5	2.15 (0.73 – 6.32)	2.25 (0.75 – 6.73)
Pancreas	6/5	1.28 (0.39 – 4.22)	1.44 (0.43 – 4.84)
Gallbladder	1/2	0.53 (0.05 – 5.89)	1.25 (0.08 – 20.36)
Larynx	5/2	2.68 (0.52 – 13.84)	4.41 (0.49 – 39.98)
Bladder	1/5	0.21 (0.02 – 1.82)	0.27 (0.03 – 2.44)
Kidney	2/1	2.14 (0.19 – 23.61)	2.08 (0.19 – 23.37)
Prostate	1/2	0.53 (0.05 – 5.89)	1.94 (0.12 – 32.42)
Uterus	7/3	2.50 (0.64 – 9.71)	3.84 (0.79 – 18.73)
Appendix	2/2	1.07 (0.15 – 7.59)	2.31 (0.20 – 25.98)
Lymphoma	5/3	1.78 (0.42 – 7.49)	4.34 (0.48 – 39.41)
Digestive tract	43/30	1.56 (0.97 – 2.51)	1.58 (0.95 – 2.64)
Digestive system	59/39	**1.67 (1.10 – 2.53)**	**1.79 (1.14 – 2.79)**
Urinary system	4/8	0.53 (0.16 – 1.77)	0.79 (0.22 – 2.88)
Hematological system	6/6	1.07 (0.34 – 3.32)	1.80 (0.42 – 7.65)
Gynecology	9/5	1.93 (0.64 – 5.79)	3.34 (0.89 – 12.53)
≥ 2 histories^#^	5/11	0.48 (0.17 – 1.39)	0.76 (0.24 – 2.47)
All sites^#^	106/70	**1.71 (1.25 – 2.36)**	**2.13 (1.49 – 3.04)**

*Adjusted for age, age at menarche, childbearing, menopause status; ^#^family history of breast cancer was excluded; bold data reflected P < 0.05.

In univariate analysis (Table 2), a significant increase in breast cancer was only observed in subjects with a family history of esophagus cancer (OR: 2.79, 95% CI: 1.16 – 6.71), digestive system cancer (OR: 1.67, 95% CI: 1.10 – 2.53), and any other cancer (OR: 1.71, 95% CI: 1.25 – 2.36).

When age, age at menarche, childbearing, and menopause status were adjusted (Table 2), a significant increase in breast cancer was still observed in subjects with a family history of esophagus cancer (OR: 2.70, 95% CI: 1.11 – 6.57), digestive system cancer (OR: 1.79, 95% CI: 1.14 – 2.79) and any other cancer (OR: 2.13, 95% CI: 1.49 – 3.04). Additionally, a significantly increased breast cancer risk was observed with a family history of lung cancer (OR: 2.49 95% CI: 1.10 – 5.65). No significant increase in breast cancer was observed with a family history of cancer in other systems (all *P* > 0.05). Furthermore, no significantly increased breast cancer risk was observed in subjects with two or more family histories of other cancers (OR: 0.76, 95% CI: 0.24 – 2.47).

In subgroup analysis (Table 3), a significant increase in both hormone receptor positive (OR: 2.36, 95% CI: 1.60 – 3.47) and negative (OR: 2.29, 95% CI: 1.38 – 3.78) breast cancer was observed with a family history of any other cancer. Importantly, a significant increase in hormone receptor positive breast cancer was observed with a family history of lung cancer (OR: 3.37, 95% CI: 1.45 – 7.82); however, subjects with a family history of lung cancer did not show a significant increase in hormone receptor negative breast cancer (OR: 1.30, 95% CI: 0.33 – 5.14). Additionally, a significant increase in hormone receptor negative breast cancer was observed in subjects with a family history of esophagus cancer (OR: 6.19, 95% CI: 2.30 – 16.71) and uterus cancer (including endometrial and cervical cancers, OR: 6.92, 95% CI: 1.12 – 42.89), and no significant increase in hormone positive breast cancer was observed. Similarly, a significant increase in hormone receptor negative breast cancer was observed with a family history of digestive tract cancer (OR: 2.05, 95% CI: 1.03 – 4.10) and gynecology cancer (OR: 6.79, 95% CI: 1.46 – 31.65), and no significant increase in hormone positive breast cancer was observed. Additionally, a significant increase in both hormone receptor positive (OR: 1.79, 95% CI: 1.09 – 2.93) and negative (OR: 2.29, 95% CI: 1.26 – 4.17) breast cancer was observed with a family history of digestive system cancer.

**Table 3:** **Subgroup analysis of breast cancer risk according to family history of selected cancers**

Site	HR positive*		HR negative*		≤ 50 y*		> 50 y*	
	Cases/control (n/n)	OR (95% CI)	Cases/control (n/n)	OR (95% CI)	Cases/control (n/n)	OR (95% CI)	Cases/control (n/n)	OR (95% CI)
breast	12/16	1.69 (0.69 – 4.17)	**9/16**	**3.59 (1.36 – 9.47)**	12/16	2.23 (0.81 – 6.14)	14/16	2.43 (0.73 – 8.20)
Lung	**17/13**	**3.37 (1.45 – 7.82)**	4/13	1.30 (0.33 – 5.14)	11/13	2.01 (0.70 – 5.79)	10/13	3.24 (0.85 – 12.42)
Esophagus	7/7	1.56 (0.53 – 4.55)	**11/7**	**6.19 (2.30 – 16.71)**	10/7	2.64 (0.81 – 8.66)	8/7	2.50 (0.65 – 9.67)
Stomach	15/17	1.69 (0.80 – 3.60)	4/17	0.65 (0.18 – 2.39)	8/17	1.10 (0.40 – 3.03)	11/17	1.49 (0.51 – 4.32)
Intestine	6/8	1.21 (0.37 – 3.95)	1/8	0.55 (0.06 – 4.68)	4/8	0.72 (0.15 – 3.36)	3/8	1.16 (0.22 – 6.11)
Liver	6/5	2.29 (0.68 – 7.70)	4/5	2.95 (0.74 – 11.84)	4/5	1.66 (0.37 – 7.53)	6/5	2.82 (0.55 – 14.58)
Pancreas	4/5	1.53 (0.40 – 5.84)	2/5	1.57 (0.28 – 8.66)	4/5	5.07 (0.56 – 45.84)	2/5	0.51 (0.09 – 2.91)
Gallbladder	1/2	2.14 (0.13 – 35.41)	/	/	/	/	/	/
Larynx	4/2	5.19 (0.53 – 50.70)	/	/	/	/	2/2	1.83 (0.16 – 20.55)
Bladder	1/5	0.41 (0.05 – 3.71)	/	/	1/5	0.39 (0.04 – 3.81)	/	/
Kidney	2/1	3.12 (0.28 – 35.11)	/	/	2/1	1.93 (0.17 – 21.76)	/	/
Prostate	1/2	2.78 (0.17 – 46.34)	/	/	/	/	/	/
Uterus	4/3	3.23 (0.58 – 17.98)	**3/3**	**6.92 (1.12 – 42.89)**	2/3	1.11 (0.15 – 8.11)	/	/
Appendix	1/2	1.83 (0.11 – 30.22)	1/2	6.01 (0.36 – 101.25)	/	/	1/2	0.99 (0.06 – 16.34)
Lymphoma	4/3	5.26 (0.54 – 51.20)	1/3	4.64 (0.28 – 78.24)	2/3	1.37 (0.09 – 22.14)	/	/
Digestive tract	27/30	1.57 (0.89 – 2.77)	**16/30**	**2.05 (1.03 – 4.10)**	22/30	1.49 (0.74 – 3.00)	21/30	1.63 (0.77 – 3.49)
Digestive system	**37/39**	**1.79 (1.09 – 2.93)**	**22/39**	**2.29 (1.26 – 4.17)**	**31/39**	**1.88 (1.03 – 3.43)**	28/39	1.67 (0.86 – 3.25)
Urinary system	4/8	1.19 (0.33 – 4.31)	/	/	4/8	1.12 (0.27 – 4.58)	/	/
Hematological system	4/6	1.78 (0.35 – 8.94)	2/6	3.02 (0.48 – 18.88)	3/6	0.75 (0.12 – 4.64)	/	/
Gynecology	5/5	2.78 (0.65 – 11.87)	**4/5**	**6.79 (1.46 – 31.65)**	3/5	1.74 (0.28 – 10.66)	6/5	6.16 (0.73 – 52.15)
Two or more histories^#^	4/11	0.96 (0.27 – 3.39)	1/11	0.58 (0.07 – 4.92)	/	/	5/11	1.54 (0.35 – 6.65)
All sites^#^	**73/70**	**2.36 (1.60 – 3.47)**	**32/70**	**2.29 (1.38 – 3.78)**	**58/70**	**1.92 (1.21 – 3.05)**	**48/70**	**2.39 (1.36 – 4.20)**

*HR*, Hormone receptor; *Adjusted for age, age at menarche, childbearing, menopause status; ^#^family history of breast cancer was excluded; bold data reflected P < 0.05.

Importantly, a significantly increased breast cancer risk was observed with a family history of any other cancer for women both younger (OR: 1.92, 95% CI: 1.21 – 3.05) and older (OR: 2.39, 95% CI: 1.36 – 4.20) than 50 y (Table 3). There was no significantly increased breast cancer risk with a family history of any single cancer for subjects older than 50 y or 50 y and younger. Interestingly, a significant increase in breast cancer was observed with a family history of digestive system cancer for subjects 50 y and younger (OR: 1.88, 95% CI: 1.03 – 3.43) not for subjects 50 y older (OR: 1.67, 95% CI: 0.86 – 3.25) (Table 3).

## Discussion

In this hospital-based case-control study, the risk of breast cancer was increased in subjects with a family history of esophagus cancer, lung cancer and digestive system cancer in first-degree relatives. In subgroup analysis, the risk of hormone receptor positive breast cancer was increased in subjects with a family history of lung cancer, while the risk of hormone receptor negative breast cancer was increased in subjects with a family history of esophagus cancer, uterus cancer, and gynecology cancer. Additionally, a significant increase in breast cancer was observed with a family history of digestive system cancer for subjects 50 y and younger, not for subjects 50 y older.

Previous studies have reported that family history of colon, prostate, ovarian cancers has been associated with an increased breast cancer risk [9, 19]. However, Negri E and colleagues [8] have found that there was no material association with family history of cancer in general, excluding breast cancer. Another previous study [27] have found association between breast and prostate cancers. In this study, we firstly found that breast cancer risk was increased in subjects with a family history of lung cancer and esophagus cancer. Importantly, the risk of breast cancer was also increased in subjects with a family history of digestive system cancer in this study. Turati F and colleagues [28] found a significant association between breast cancer and family history of colorectal cancer with a OR of 1.5. Lifestyle change and no healthy lifestyles may contribute to increasing digestive system cancer morbidity in developing countries. Breast cancer and digestive system cancer may share some etiological factors, including increased body mass index, low fruit and vegetable intake, smoking, and low energy intake [29–31]. Our findings and previous studies suggest that healthy lifestyles, including high dietary fiber, increased physical activity, and no smoking, should be kept [32]. Negri E and colleagues [8] have reported that an unexpected relation of breast cancer risk with family history of gallbladder cancer, based on only 7 cases and 1 control. They have inferred that this association may be due simply to chance, since the lower confidence limit was around 1. Turati F and colleagues [28] found a significant association between breast cancer and family history of hemolymphopoietic cancers. In this study, no relation of breast cancer risk with family history of gallbladder cancer and lymphoma was observed. Our findings confirm the conclusion of the previous studies. However, there was an unexpected relation of breast cancer risk with family history of lung cancer and esophagus cancer, and these associations could not be due simply to chance in this study. Nutritional and diet factors may contribute to these associations. Moreover, ethnicity may contribute to the disparity of our results with the previous studies. Future studies in our country should be taken to confirm our results.

The relationships between having a family history of breast cancer and risk of subtypes of breast cancer defined by hormone receptor status are inconsistent [18, 19, 33], and the relationship between having a family history of other cancers and risk of subtype of breast cancer has not been reported. An increased risk of hormone receptor negative breast cancer was observed in subjects with a family history of esophagus cancer, uterus cancer, digestive tract cancer, and gynecological cancer; however, no significantly increased risk of hormone receptor positive breast cancer was observed in subjects with a family history of above cancers. Our findings suggest that hormone receptor positive and negative breast cancers have different genetic components. The presence of mutations in the BRCA1 gene are associated with hormone receptor negative breast cancer [34]. Importantly, BRCA1/2 genes mutations have been detected in gynecological cancer and digestive system cancer [35–40]. BRCA1/2 genes mutations may be contributed to the relationship between having a family history of gynecological cancer and digestive system cancer and increased risk of hormone receptor negative breast cancer. Besides, other gene mutations may also be contributed to the relationship, and should be detected in future studies.

Jiang and colleagues find that women with a family history of breast cancer were more likely to have hormone receptor negative breast cancer than women without a family history, and the association was limited to cancers diagnosed before age 50 [33]. Similarly, we found a significant increase in breast cancer was observed with a family history of digestive system cancer for subjects 50 y and younger not for subjects 50 y older. Prior estimates have suggested more parts of women diagnosed with breast cancer before age 50 years carry germ-line mutations in BRCA1 than women after age 50 years [41]. The difference on this issue may also be attributed to the presence of gene mutations.

Our study was the systematic investigation of all other cancers in first-degree relatives and the subsequent breast cancer risk. The strengths of this study are described as following. First, previous studies suggest that the recall of cancer in second-degree is less reliable [8, 42]. Only first-degree relatives were included in our study to reduce recall bias. Second, cases of breast cancer may tend to recall a family history of other cancers more accurately than controls [8], and the interviews of family history for all cases and controls were performed before diagnose by trained interviewers. Third, the pathology of all cases and controls was confirmed by an experienced pathologist. Benign breast disease was selected as controls, which included fibroadenoma, mastopathy, intraductal papilloma, and others. The association between breast cancer risk and family history of other cancers may be underestimated; however, no major recall bias existed in the present case-control study.

However, several potential limitations of this study should be considered when interpreting these results. First, the information on other potential risk factors of breast cancer such as diet, smoking, body mass index, and number of siblings, was unavailable. Some of the significant associations could be attributed to chance. Moreover, number of first degree relatives [43] was not adjusted in this study due to small number of cases ≥ 2 first degree relatives (Table 2). Second, age at diagnosis of the affected relatives was not collected, so the relationship between breast cancer risk and age at diagnosis of the affected relatives could not be calculated. Third, due to the nature of the design of this study, our findings should be confirmed by future cohort studies that also considering other risk factors for breast cancer. Forth, the number of incident cases from some neoplasms was relatively small, and this limited the precision of the risks estimates, especially for subgroup analyses. Some associations, which may be by chance findings or others based on a limited number of exposed cases and controls, need independent confirmation.

## Conclusions

In conclusion, a family history of esophagus cancer, lung cancer, and digestive system cancer in first-degree relatives was directly associated with breast cancer risk. In subgroup analysis, the influence of having a family history of other cancers seems more likely to be limited to hormone receptor negative breast cancer. An increased breast cancer risk was observed in subjects 50 y and younger with a family history of digestive system cancer in first-degree relatives. Future cohort studies with a larger sample are still needed to confirm our findings and consider other risk factors.

## Abbreviations

CI: Confidence interval
ER: Estrogen receptor
OR: Odds ratio
PR: Progesterone receptor
SD: Standard deviation.
